# A population-based registry as a source of health indicators for rare diseases: the ten-year experience of the Veneto Region’s rare diseases registry

**DOI:** 10.1186/1750-1172-9-37

**Published:** 2014-03-19

**Authors:** Monica Mazzucato, Laura Visonà Dalla Pozza, Silvia Manea, Cinzia Minichiello, Paola Facchin

**Affiliations:** 1 Rare Diseases Coordinating Center, Rare Diseases Registry, Veneto Region, Padua, Italy

**Keywords:** Rare diseases, Registry, Epidemiology, Public health indicators

## Abstract

**Background:**

Although rare diseases have become a major public health issue, there is a paucity of population-based data on rare diseases. The aim of this epidemiological study was to provide descriptive figures referring to a sizable group of unrelated rare diseases.

**Methods:**

Data from the rare diseases registry established in the Veneto Region of north-east Italy (population 4,900,000), referring to the years from 2002 to 2012, were analyzed. The registry is based on a web-based system accessed by different users. Cases are enrolled by two different sources: clinicians working at Centers of expertise officially designated to diagnose and care patients with rare diseases and health professionals working in the local health districts. Deaths of patients are monitored by Death Registry.

**Results:**

So far, 19,547 patients with rare diseases have been registered, and 23% of them are pediatric cases. The overall raw prevalence of the rare diseases monitored in the population under study is 33.09 per 10,000 inhabitants (95% CI 32.56-33.62), whilst the overall incidence is 3.85 per 10,000 inhabitants (95% CI 3.67-4.03). The most commonly-recorded diagnoses belong to the following nosological groups: congenital malformations (Prevalence: 5.45/10,000), hematological diseases (4.83/10,000), ocular disorders (4.47/10,000), diseases of the nervous system (3.51/10,000), and metabolic disorders (2,95/10,000). Most of the deaths in the study population occur among pediatric patients with congenital malformations, and among adult cases with neurological diseases. Rare diseases of the central nervous system carry the highest fatality rate (71.36/1,000). Rare diseases explain 4.2% of general population Years of Life Lost (YLLs), comparing to 1.2% attributable to infectious diseases and 2.6% to diabetes mellitus.

**Conclusions:**

Our estimates of the burden of rare diseases at population level confirm that these conditions are a relevant public health issue. Our snapshot of their epidemiology is important for public health planning purposes, going to show that population-based registries are useful tools for generating health indicators relating to a considerable number of rare diseases, rather than to specific conditions.

## Background

Rare diseases have become a topical issue in the medical and societal debate. After being ignored for some time, this topic has been attracting increasing attention and come to be recognized as a priority to consider both in research programs and in health policy implementation [1].

Rare diseases often reportedly have a considerable impact on the health of a community in terms of the related impairments, long-term disabilities and shorter life-expectancy, as well as high human and social costs [2,3]. Although more attention has been addressed to this issue of late, only a few population-level snapshots of the impact of rare diseases are currently available. It has been estimated, for example, that rare diseases are responsible for about one in three cases of severe impairment in children [4]. There is a general shortage of epidemiological data on many rare diseases, making it impossible to calculate the true burden of these conditions as a whole in terms of years of life lost due to premature death, those lost due to disability, and so on [5]. The paucity and fragmentation of available data also make it difficult to compare the burden attributable to rare diseases with the one due to other more common diseases, injuries or risk factors, and such comparisons are fundamental to health care decision-making and planning at population level [6,7].

Despite the urgent need for reliable data, the epidemiological figures on rare diseases are objectively difficult to collect for a number of reasons.

The first issue concerns how to clearly establish which diseases should be defined as rare. Between 5,000 and 8,000 rare diseases are believed to exist, but these figures are continuously being adjusted as new forms come to light [8]; and several different definitions of rare disease are in use around the world [9-13].

The second problem is how to trace patients with rare diseases in public health information systems. The International Classification of Diseases (ICD) [14] has some limitations when it comes to coding and classifying most rare diseases, and consequently in identifying the patients involved [15]; just to give an example, ICD-10 provides a specific code for less than 250 rare diseases [16]. In the context of the ongoing ICD revision process, a specific Topic Advisory Group has been established for rare diseases, with a view to improving the coding and classification of these conditions [17]. Orphanet coordinates this activity, and has also developed an inventory of rare diseases. A unique identifier, called ORPHA number, is assigned to each rare disease [18]. In France, one of the key actions of the second French Plan for rare diseases was put in place: ORPHA numbers have entered in use in hospital information systems, allowing a better traceability of rare diseases patients’ into the healthcare system. Of course, the effect of this effort will be observable only in some years from now.

For all the above reasons, the availability of reliable epidemiological data on rare diseases is increasingly perceived as a strong and urgent need. At European level, the Council Recommendation on an action in the field of rare diseases, issued in 2009, has recognized the importance of supporting specific disease information networks, registries and databases [19]. The attention of all the interested parties involved (researchers, patients with rare diseases, governmental bodies, etc.) is focusing on choosing the best methods for systematically collecting data on rare disorders. Some countries have taken steps to develop platforms to facilitate the collection of disease-specific data. In the USA, a movement involving both researchers and patients supported the development of a global rare diseases registry for collecting a considerable amount of information on potentially thousands of diseases and linking these data with bio-repositories [20,21].

On the European front, Orphanet periodically provides a snapshot of the data being collected on rare diseases by 588 databases and registries [22]; the vast majority of the sources listed by Orphanet are maintained by public institutions (academia), while very few involve governmental bodies. They differ in terms of data sources, structure, tools used for data collection and coverage.

An important distinction has to be made between patient databases and registries. Patient databases are not designed to provide a full coverage of the population, so they cannot be used to estimate the prevalence/incidence of the diseases monitored. Such figures can only be obtained from the data in registries, a prerequisite of which is to define an accurately-monitored catchment area in which all registered cases arise (usually a region or a country) [23]. Many population registries have been set up to gather information on the epidemiology of certain rare diseases. They usually focus on single conditions or groups of diseases, such as congenital malformations [24]. Though they are important, such data registries are challenging to establish and maintain, and it is difficult to judge their ability to describe the epidemiology and global burden of rare diseases at population level [25].

In 2011, the European Commission launched the EPIRARE project with a view to establishing the requirements for rare disease registries and databases, to exploring the feasibility of defining a dataset shared by different rare disease registries, and to developing a common platform for the exchange of data. More recently, EUCERD issued a set of recommendations concerning the registration of rare diseases and the related data collection, according to which rare disease registries should be organized around population health needs and/or single rare diseases, or groups of rare diseases, rather than around their treatments [26].

In Europe, the crucial need for health indicators on rare diseases has been recognized particularly as concerns two aspects: to assess the health status and health outcomes of patients, and to monitor the efficacy of health policies and initiatives addressed to rare diseases. The use of data from registries dedicated to one or more rare diseases has been identified as strategically important to ensure the availability of many such health indicators [27]. In practice, this can only be done for some rare diseases, for which good-quality data collection schemes are already in place. A more comprehensive approach is required, however, to produce indicators that refer - if not to the whole “universe” of rare diseases - at least to a significant proportion of them. To achieve this goal, it is mandatory to adopt a population-based approach, even though this is usually considered very difficult and expensive.

The Italian rare diseases scenario has something to contribute to the debate on this complex issue. Here we describe the experience gained in this field by the Veneto Region’s rare diseases registry (VR-RDR) in the north-east of Italy. A multi-source web-based information system has been developed that combines aspects of a population-based registry (an essential source of epidemiological data for supporting health planning) with aspects of a more clinically-oriented registry (collecting data that are useful in the clinical decision-making process). The registry was implemented in the Veneto Region in 2002 and has since then been adopted successfully in other Italian regions (Trentino-Alto Adige, Emilia-Romagna, Liguria and, more recently, Campania, Apulia, Umbria and Sardinia).

The main aim of the present study is to describe the epidemiological figures relating to a group of rare diseases in the Veneto Region emerging from this first Italian population-based registry dedicated to rare diseases and covering the years from 2002 to 2012, particularly as concerns prevalence, incidence, mortality, fatality rate, and years of life lost.

## Methods

### Italian legislation on rare diseases

The Italian health-care system is a universal, regionally-based public system. A law defining Italian policy on rare diseases was issued in 2001 [28]. The key elements of this legislation are: the establishment of a list of rare diseases, the identification of regional/inter-regional Centers of expertise for rare diseases responsible for patient diagnosis and follow-up, and the creation of area-based rare disease registries.

### The list of rare diseases

The list of rare diseases attached to the Italian Law establishes which patients are entitled to benefits and facilitated access to care. The list contains 331 single diseases or groups of disorders divided into 14 nosological categories, based on the ICD9-CM [see Additional file 1]. For the groups, only a few examples of the relevant diseases are mentioned in the law. As a preliminary step, the medical team developing the registry properly identified all the diseases to consider (for which patients are eligible for benefits) in the light of the medical literature and existing databases. This also involved dealing with synonyms and eponyms. In addition, corresponding codes as used in international classifications (i.e. ICD9-CM, ICD-10, MIM and ORPHA-code) were assigned to each disease. The resulting list is continuously updated as new forms are described and new classifications are adopted. Considering the diseases included in all the groups and the disease sub-types, nearly 3,000 conditions have been identified, although some diseases, or groups of diseases, that qualify as rare are not currently covered by our monitoring system. Excluding rare cancers (which are recorded in the regional cancer registry), the conditions monitored by the rare disease registry represent 58% of all the rare diseases included in the Orphanet list.

### The care network

In 2002 the Veneto Regional Authority officially identified the Centers dedicated to the diagnosis and treatment of patients with rare diseases. In 2004 a formal collaboration agreement between four neighboring regions and provinces in the north-east of Italy (including the Veneto) led to the identification of a shared inter-regional network of Centers of expertise for specific groups of rare diseases (e.g. rare hematological conditions, rare neurological disorders, etc.); each Center has at least one clinical ward. These Centers were identified officially on the grounds of indicators and criteria established by the Regional Health Authorities signing the collaboration agreement and they are monitored continuously. The Centers of expertise are closely linked to the territorial network of public health services providing primary and specialized care, as well as non-medical services for patients with rare diseases. All health care providers involved in caring for patients with rare diseases use a common information system (IS) to share clinical data and support the delivery of benefits and services to patients. At the same time, the system provides the foundations for an area-based registry recording data on patients with rare diseases.

## The information system (IS)

### Contents

The information system connects the Centers of expertise, located at 12 different hospitals, via a protected network (*Regional Health Network Intranet*) to all the local public health authorities and all the local and hospital pharmaceutical services in the Veneto Region. This computerized system is a complex platform with a Java-based web browser application that populates a single, central Oracle database with a three-level architecture capable of collecting and managing large amounts of data. L-DAP has been implemented to manage access by encrypted users with different personal profiles.

The system collects a set of patients’ socio-demographic details, such as name, date and place of birth, gender, fiscal code, place of residence and, where applicable, place and time of death. The core element in the information system is the diagnosis of a rare disease included in a list shared by all users and regularly updated, as mentioned earlier. Specific forms have been developed over the years to manage the drug and dietary prescriptions, medical devices and prosthetics provision, and to collect each patient’s medical history. Several technical solutions have been developed to ensure a high-quality data input (with the completion of mandatory fields, data validation by checks on data format and plausibility, dropdown lists, provisional and final data-saving options, etc.). Reported errors or modifications are managed centrally. Double entries are prevented by the system, except when the same patient is diagnosed with two different rare diseases.

### Users

To access the system, every user is assigned a personal user name and password. The system assures a high standard of security. It is accessible to users via a standard browser using encrypted log-in sessions, in compliance with the Italian legislation on personal data protection [29].

The system is accessible to: (1) clinicians working at the Centers of expertise who input patients’ data (demographic details, diagnosis, prescribed treatments, clinical data, etc.); (2) health-care providers working in the primary care setting (local public health districts), who can view information they are entitled to see and input patient data that are monitored by the Centers of expertise located outside the Veneto and collaborating regions; (3) pharmacists working in the local health districts and in hospitals who view prescriptions directly and issue medications to patients according to the treatment plan formulated by clinicians at the Centers of expertise; (4) other clinicians working in hospitals and directly involved in the patients’ care.

For the moment, general practitioners do not have access to the system for security reasons (because most GPs do not have direct access to the protected regional intranet system). There are plans to find technical solutions in the near future to enable them to connect to the platform and share information with the clinicians at the Centers of expertise in charge of their patients.

All users received training before the system was implemented and updates when new modules were added to the core system. A help line is managed by trained registry personnel and can be contacted to deal with any questions.

Currently, more than 3,484 health-care professionals access the system (1,224 in the Veneto Region, and 2,260 in the other regions where the system is used); those in the Veneto Region include 508 clinicians working at the Centers of expertise, 450 users at the local public health districts, and 233 pharmacists.

### The patient’s pathway

In practical terms, a patient is referred to a given Center of the network for a complete assessment (which is free of charge when a rare disease is suspected). If a rare disease is diagnosed, this implicitly involves the clinician at the Center issuing a certificate and, at the same time, the local public health authority of the patient’s place of residence issuing an exemption document. Patients can thus receive the benefits to which they are legally entitled, including specific drugs or medical devices indicated in the treatment plan drawn up at the Center of expertise. In this way, the case is registered in the information system, thus providing the foundations for an area-based registry of patients with rare diseases.

### Setting

The population monitored includes all residents in the Veneto Region, i.e. a population of 4,853,657 as at 2012 (source: Italian National Institute of Statistics).

Eligible cases are all patients diagnosed with one of the rare diseases on the list in the Italian law (see Additional file 1) and registered in the information system from May 1, 2002 to December 31, 2012.

Cases are identified using two possible alternative sources of input data, i.e. the clinicians working at the Centers of expertise in the Veneto and the other regions sharing the same IS, or health professionals working at the local public health districts who input data on patients diagnosed and followed up by Centers of expertise outside the inter-regional area being monitored. This ensures a good coverage of the Veneto’s resident population.

### Other data sources and indicators

To ensure that almost all patients diagnosed with one of the monitored diseases are enrolled in the registry, cross-referencing with other regional data sources is done at regular intervals. These other sources include the registry of hospital discharge records, the birth registry, the records of outpatient rehabilitation services, and the death registry.

The following indicators were calculated with a 95% confidence interval from registry data, by age group (0–12 months; 1–14 years, 15–17 years; 18–64 years; over 65 years old) and by nosological group: prevalence, incidence, mortality rate and fatality rate. Here we report the results for the interval between 1 May 2002 and 31 December 2012, estimating the point prevalence as at 31 December 2012.

To calculate the years of life lost (YLLs) we considered life expectancies by age and gender based on life tables by age and gender referring to the Veneto population (source: Italian National Institute of Statistics, year 2010).

Our statistical analysis was performed using the SAS package, rel. 9.1 (SAS Institute Inc., Cary, NC, USA).

## Results

During the period from May 2002 through December 2012, there were 19,547 individuals diagnosed with one of the rare diseases listed in the Italian Law (see Additional file 1) among the population monitored. Another 4,405 patients were diagnosed at Centers of expertise in the Veneto Region, but lived outside the study area, yielding a total of 23,952 cases recorded in the registry, and 1,424 different rare diseases diagnosed; 56 individuals had more than one rare disease.

Of the 19,547 patients who lived in the study area, 52% were female and 48% male. The registered patients were a mean 38 years old. Of the patients alive as at 31 December 2012, 23% were under 18 years of age. The overall raw prevalence of rare diseases in the study population was 33.09 per 10,000 inhabitants (95% CI 32.56-33.62) (Table 1).

**Table 1:** Prevalence, incidence, mortality and fatality rates by age group

	**Prevalence per 10,000**		**Crude incidence per 10,000**		**Mortality per 100,000**		**Fatality per 1,000**	
Age	Rate	C.I. 95%	Rate	C.I. 95%	Rate	C.I. 95%	Rate	C.I. 95%
**0-17 years**	44.05	42.61-45.49	3.96	3.52-4.39	10.21	8.01-12.41	3.59	1.37-5.81
0-12 months	25.73	21.23-30.24	25.73	21.23-30.24	0.56*	0.35*-0.77*	19.23	4.74-33.72
1-17 years	45.22	43.71-46.72	4.26	3.80-4.73	7.29	6.69-7.90	2.98	0.92-5.04
*1-14 years*	*46.25*	*44.58-47.93*	*4.41*	*3.89-4.92*	*8.00*	*7.30-8.69*	*3.10*	*0.81-5.39*
*15-17 years*	*40.26*	*36.84-43.68*	*3.57*	*2.55-4.59*	*3.93*	*2.86-5.00*	*2.36*	*0.00-6.99*
**18-64 years**	31.34	30.72-31.96	3.70	3.48-3.91	9.03	7.97-10.09	3.70	2.33-5.07
**over 65 years**	17.68	16.83-18.53	2.90	2.56-3.25	28.51	25.10-31.91	26.79	17.49-36.10
**TOTAL**	**33.09**	**32.56-33.62**	**3.85**	**3.67-4.03**	**13.04**	**12.02-14.06**	**5.99**	**4.58-7.40**

Veneto Region’s rare diseases registry. * rate per 1,000 live births.

The prevalence among infants under 1 year old was 25.73 per 10,000 newborns (95% CI 21.23-30.24), with an overall prevalence in pediatric patients (0 to 17 years old) of 44.05 per 10,000 (95% CI 42.61-45.49). The prevalence of rare diseases was 31.34 and 17.68 per 10,000 inhabitants in the 18–64 and ≥65-year-old age groups, respectively.

The overall raw annual incidence of rare diseases was 3.85 per 10,000 inhabitants (95% CI 3.67-4.03), corresponding to nearly one new case a year for every 10 prevalent cases (Table 1). The incidence in 1- to 17-year-olds was 4.26/10,000 (95% CI 3.80-4.73). The annual incidence rates for the 18- to 64- and the ≥65-year-olds were 3.70 and 2.90 per 10,000 inhabitants, respectively.

The annual raw mortality rate for patients with rare diseases was 13.04 per 100,000 inhabitants (95% CI 12.02-14.06). The overall infant mortality rate was 0.56 per 1,000 live births (*vs* 2.8 in the general population), and the neonatal mortality rate was 0.16 per 1,000 live births (*vs* 2.2 in the general population). As for the post-neonatal mortality rate, this was 0.4 per 1,000 live births in the monitored population, two thirds of the rate observed in the general population (0.6 per 1,000 live births). The overall mortality rate was 7.29 per 100,000 (95% CI 6.69-7.90) among the 1- to 17-year-olds, 9.03 for the 18- to 64-year-olds, and 28.51 for the ≥65-year-olds.

**Figure 1:**
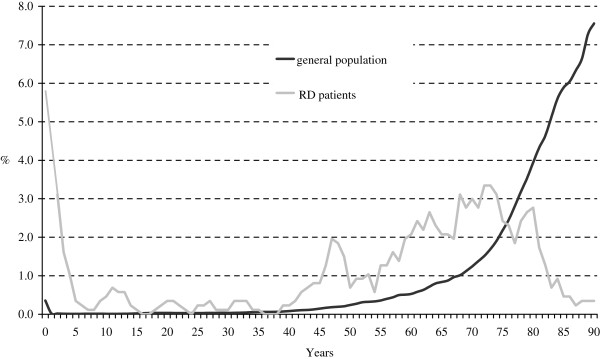
**Age at death in patients with rare diseases and in the general population.** Veneto Region’s rare diseases registry, Italian National Institute of Statistics (ISTAT).

When age at death was compared between rare disease patients and the general population, the former were likely to die mainly in older age classes (but earlier than members of the general population) and in the first year of life (Figure 1).

As concerns life expectancy, deaths in the rare disease population accounted for 14,558 years of life lost, corresponding to 4.2% of the total years of life lost by the general population - nearly four times as many as the YLLs due to infectious diseases (1.2%), and nearly twice as many as the YLLs due to diabetes (2.6%).

The overall annual raw fatality rate was 5.99 per 1,000 (95% CI 4.58-7.40) (Table 1). As expected, the highest fatality rates were seen for infants aged <1 year (19.23 per 1,000) and cases ≥65 years old (26.79 per 1,000).

Table 2 shows the incidence, prevalence, mortality and fatality rates by nosological group according to the ICD9-CM.

**Table 2:** Prevalence, incidence, mortality and fatality rates by nosological group

**GROUPS OF DISEASES (ICD9-CM)**	**Prevalence per 10,000**	**Incidence per 10,000**	**Mortality per 100,000**	**Fatality per 1,000**
Infectious and parasitic diseases	0.05	0.002	-	-
Neoplasms	1.37	0.103	0.24	3.90
Endocrine disorders	1.05	0.132	0.13	2.25
Disorders of amino-acid transport and metabolism	0.61	0.041	0.27	8.64
Disorders of carbohydrate transport and metabolism	0.22	0.027	0.03	2.14
Disorders of lipoid metabolism	0.22	0.025	0.13	10.13
Disorders of mineral metabolism	1.65	0.213	0.21	2.51
Disorders of plasma protein metabolism	0.06	0.002	0.16	38.99
Metabolic disorders (others)	0.19	0.021	0.21	19.52
Immunity disorders	1.23	0.118	0.24	3.30
Diseases of the blood and blood-forming organs	4.83	0.445	1.18	4.21
Central nervous system disorders	1.79	0.176	5.64	71.36
Peripheral nervous system disorders	1.72	0.223	0.77	8.68
Disorders of the eye and adnexa	4.47	0.759	0.45	2.12
Diseases of the circulatory system	1.12	0.103	0.59	14.06
Diseases of the digestive system	0.63	0.070	0.21	6.03
Diseases of the genitourinary system	0.19	0.037	0.05	8.40
Diseases of the skin and subcutaneous tissue	1.20	0.215	0.85	19.11
Diseases of the musculoskeletal system and connective tissue	2.76	0.250	0.61	3.98
Congenital anomalies	5.45	0.621	0.96	3.21
Certain conditions originating in the perinatal period	0.03	0.002	0.05	19.36

Veneto Region’s rare diseases registry.

**Figure 2:**
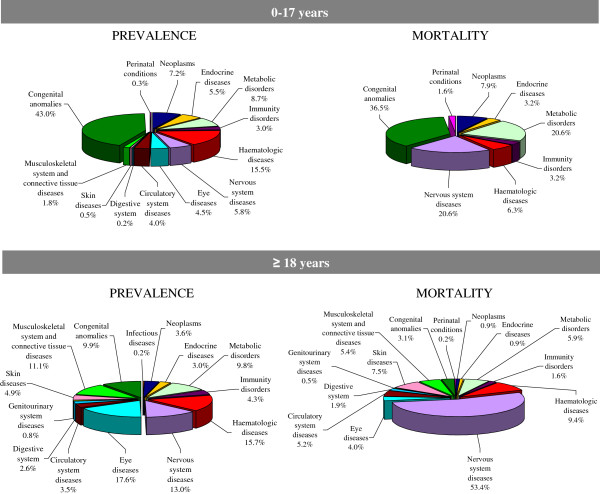
**Percentage distribution of prevalence and mortality rates by nosological group.** Veneto Region’s rare diseases registry; cases 0–17 years *vs* ≥ 18 years old.

The nosological groups with the highest prevalence were congenital malformations (5.45/10,000), hematological diseases (4.83/10,000), and eye disorders (4.47/10,000), followed by diseases of the nervous system (3.51/10,000), and metabolic disorders (2.95/10,000). The distribution by nosological group and age clarifies their different contribution to the burden of pediatric and adult disease (Figure 2).

Congenital malformations were involved in 43% of all rare disease patients under 17 years old as opposed to 9.9% of patients ≥18 years old. Most of the children diagnosed with congenital malformations had complex syndromes (36.5% of cases) or chromosomal anomalies (28.3%); a non-negligible proportion of them had congenital anomalies involving the digestive system (11.2%). One in two patients ≥18 years old had complex syndromes (23.2%) or chromosomal anomalies (29.7%), as in the pediatric population. A sizable proportion of patients had malformations involving the musculoskeletal system (17.8%) and skin (10.9%), i.e. epidermolysis bullosa and ichthyoses.

Hematological diseases (15.5%) formed the second largest group of 0- to 17-year-olds, due to a high prevalence of thalassemia and sickle cell disease in the study area, the former resulting from an endemic diffusion, the latter relating to the high immigration rate in the region considered [30,31]. Hereditary metabolic diseases were diagnosed in 8.7% of all pediatric patients. The most common sub-groups of these disorders concerned the metabolism and transportation of amino acids and carbohydrates. For 5.8% of the <17-year-old patients diagnosed with rare diseases, the problem concerned the nervous system, and most of these patients had muscular dystrophies.

The distribution of patients by nosological group changed in the adult population. The rare diseases most often encountered were eye disorders (17.6%) - mainly keratoconus and retinal diseases, followed by hematological conditions (15.7%) - particularly hereditary coagulation defects and hereditary anemias. The percentage of patients with nervous system diseases was nearly twice as high in adults as in the pediatric age group (13% vs 5.8%). Whilst peripheral and central nervous system (CNS) diseases were equally represented among pediatric patients, adults had a higher proportion of CNS diseases, amyotrophic lateral sclerosis (ALS) being the most frequent diagnosis. Interestingly, congenital malformations (9.9%) and metabolic disorders (9.8%) accounted for a non-negligible proportion of rare disease cases in the adult population.

When the mortality data for the pediatric study population were considered, congenital malformations were responsible for 36.5% of deaths, followed by metabolic disorders (20.6%) and nervous system diseases (20.6%). While these three groups of rare conditions accounted for nearly half of all the prevalent cases among pediatric patients (57.5%), they were responsible for two thirds of the deaths in children and adolescents (77.7%).

Though neurological diseases only involved 13% of all the adult patients, they were the cause of more than half of the deaths in the adult and elderly population monitored by the registry, due mainly to ALS. The very poor prognosis associated with this diagnosis explains the very high fatality rate recorded for rare CNS diseases in the population monitored (71.36/1,000) (Table 2). Some other nosological groups coincided with high fatality rates too, despite a relatively small number of patients being involved; this was the case of rare skin diseases (19.11/1,000), perinatal conditions (19.36/1,000), and rare diseases of the circulatory system (14.06/1,000). Protein metabolism disorders also carried a high fatality rate, due mainly to the large percentage of fatal cases among patients with mixed cryoglobulinemia.

Some of the most prevalent groups of diseases carried the lowest fatality rates, reflected in the long-term survival of patients with these chronic conditions.

Moreover, the Registry allows to provide, among nosological groups, the real number of cases per specific disease, classified according to ICD code, or ORPHA number or MIM number. As an example, at 31th December 2012, 79 cases of Duchenne dystrophy, 316 cases of Hemophilia A, 52 of Hemophilia B and 18 of Hemophilia C were recorded in the Registry.

## Discussion

As far as we know, the epidemiological figures reported here, derived from a population-based registry monitoring a broad group of unrelated rare conditions, provide the first indication of the magnitude of the public health problem associated with rare diseases.

The snapshot provided by this study of the impact of rare diseases on different age groups of the population shows the differences in the distribution of the various nosological entities. One in three patients with rare diseases is a pediatric case. In this age group, the most commonly seen rare diseases (congenital malformations, hematological diseases and inherited metabolic diseases) are characterized by a potentially high severity, as demonstrated by the corresponding fatality and mortality data. The epidemiological figures for the pediatric population confirm the importance of the neonatal period for early diagnosis and treatment, and for improving outcomes. Data from population-based registries that enable the numbers of cases of rare disease to be estimated are important for the purpose of optimizing the organization and functioning of the expanded neonatal screening programs as they become available [32,33]. In this evolving scenario, the value of information systems capable of supporting patients’ long-term follow-up has already been recognized, with a view to monitoring outcomes and assessing the utility of any programs implemented [34,35].

In our adult sample population, the rare diseases most often identified were eye disorders that are generally a cause of disability rather than mortality. Another considerable proportion of the adult patients had rare neurological diseases: this group accounted for half of all deaths and carried a correspondingly high fatality rate, attributable mainly to ALS. These data should orient the allocation of resources, supporting specific measures to address the health care needs of patients with these conditions [36].

Our registry data go to show that quite a lot of patients survive into adulthood, especially among those in certain nosological groups, such as congenital anomalies [37,38]. Nearly 10% of the adult patients in our sample population had complex congenital malformations, and one in two patients registered with a diagnosis of inborn errors of metabolism was over 18 years old, which indicates a longer survival than in previous studies [39,40]. We consider these findings of great interest because they have important implications in terms of the need to develop new models of health care provision, like those already adopted for some rare conditions [41-45], which will have to be extended to more and more patients with rare diseases surviving into adulthood [46].

On a European level, the production of public health indicators in the area of rare diseases, like those already generated for perinatal conditions and congenital anomalies [47,48], has been strongly advocated. Such public health indicators, derivable only from population-based registries, are particularly important for the purpose of implementing and monitoring policies specifically addressing rare diseases. The availability of epidemiological data is crucial, for example, when it comes to deciding how many Centers of expertise are needed for each type of rare disease (depending on the patients’ distribution), or to setting up and assigning functionalities to other healthcare providing networks with the particular problems posed by rare diseases in mind (e.g. rehabilitation and palliative care services).

Health indicators of extensive use, such as YLLs, are utilized to assess the burden of diseases on the general population to guide health policies and public funding. The present study highlights that rare diseases’ impact on population health is 2–4 times higher than other causes on which health policies and public funding focus at most.

When trying to obtain public health indicators on rare diseases, the advantages of using health statistics referring to large populations are limited by patient traceability problems because rare diseases are under-represented in current coding and classifications systems [49]. On the other hand, it would be too costly to establish and maintain multiple rare disease registries covering large populations. The dilemma concerning which sources to use to obtain reliable health indicators on rare diseases might be solved by a combined approach. Current statistics could be used (bearing the above-mentioned limitations in mind) to obtain estimates for basic indicators until the new ICD (in which rare diseases are more appropriately represented) has come into use. At the same time, specific systems for monitoring rare diseases already implemented in certain geographical areas and different countries could serve as data sources (so long as they are sufficiently broad-based) for the purpose of establishing specific indicators and enabling projections for the population of a country or the whole of Europe.

According to the prevalence data emerging from our study on the rare diseases considered here (3.3/1,000 inhabitants), and to the prevalence data available from other data sources (Orphanet) for unmonitored entities (3.3/1,000 inhabitants), we can estimate an overall prevalence of rare diseases of 6.6/1,000 inhabitants, which rises to 12.8-19.5/1,000, according to the other more or less conservative estimates of the prevalence of rare cancers considered [50,51].

Judging from these figures, we can assume that between 6,500,000 and 9,880,000 people living in the EU28 countries have a rare disease, which corresponds to 1.3%-2% of the whole population. This figure differs somewhat from the usually-reported estimates of 6-8% of the whole European population suffering from rare diseases [52], a difference that can probably be explained by the fact that the latter higher figures are not derived, to the best of our knowledge, from epidemiological studies conducted at population level. On the other hand, given the previously-mentioned limits of our study, the figures reported here should be considered as minimum values. In the light of the above figures, some of the concerns voiced about the financial sustainability of health policies specifically addressing patients with rare diseases in times of economic austerity need to be carefully reconsidered. Moreover, indicators of the burden of rare diseases are probably underestimated to some degree, in which case the already far from negligible impact of these conditions at population level is bound to be even greater.

### Limitations

Although this descriptive population-based study provides insight into the epidemiology of a sizable group of rare diseases being monitored by a unique web-based population registry, it has some limitations that need to be mentioned. First, the data presented here could underestimate the phenomenon because the registry may not have enrolled all the individuals living in the area monitored. This could be the case of patients with a rare disease that has yet to be diagnosed (diagnostic delays are known to be common for these rare conditions) [53,54], or patients with severe forms of disease who were not registered due to early mortality. Elderly cases may have been overlooked because their shorter life expectancy and risk of death from other diseases would make them less likely to be referred to Centers of expertise. Having said as much, we can assume that our figures might be only slightly underestimated because the registration system has been in use since 2002, because patient enrolment is linked with exemption from health care costs, and specialized diagnostic facilities are only available at Centers of expertise - all aspects that facilitate a more comprehensive patient capture. The use of multiple sources to identify cases (Centers of expertise, local public health authorities and pharmaceutical services) also helps to assure a good coverage of the population.

Another limitation may relate to the contention that clearly-defined diagnostic criteria are not available for all the rare diseases monitored. We can assume, however, that the diagnoses registered in our system are highly accurate because they are established by clinicians at Centers of expertise for rare diseases, identified on the strength of highly-standardized criteria, and routinely audited by the Regional Health Authority. Although patient registration is linked to the issue of an exemption, the quality of the clinical data collected is higher than in an administrative database [15]. Furthermore, the level of diagnostic detail adopted by the Veneto Regional registry is also higher than in the list contained in the Italian law in order to identify patients’ diagnoses as specifically as possible because the information system on which the registry is based was designed to enable clinicians at the Centers of expertise to prescribe drugs and devices on-line, draw up health care plans, and monitor patients’ major clinical events. Linking the registration process with the care dimension guarantees a good-quality data collection and its maintenance over time.

**Figure 3:**
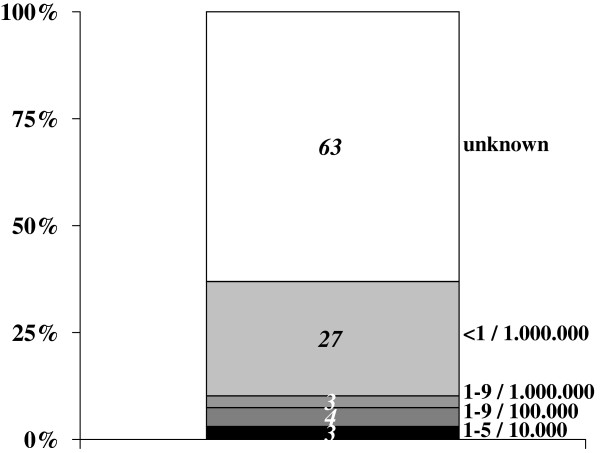
**Percentage distribution of prevalence estimates of rare diseases not currently monitored by the Veneto Region’s rare diseases registry. **Source: Orphanet Report Series - Prevalence of rare diseases: Bibliographic data - June 2013 (Reference 50)

An additional limitation of this study warrants specific consideration. The list of rare diseases monitored by the registry described here is not as exhaustive as the Orphanet one, for instance [55]. This limitation will be partially overcome in future when the Italian Government is expected to add another 109 single diseases or groups of diseases to its original list. For the time being, specific groups of diseases, such as infectious diseases, respiratory disorders, renal diseases, rheumatological diseases and rare tumors, are particularly under-represented. Some diseases (or groups of diseases), including cystic fibrosis and rare tumors, are monitored in Italy and many other European countries by dedicated registries that often have a long history of data collection. Leaving rare cancers aside, the list of rare diseases in the various groups monitored by the registry described in the present work cover the 58% of all those on the Orphanet list so, to obtain a global estimate of the burden of rare diseases, we considered prevalence data from multiple sources and referring to groups particularly under-represented in the Italian law (i.e. diseases of the respiratory system, infectious diseases, renal diseases and rheumatological diseases). The list of rare diseases for which prevalence estimates are available and updated regularly by Orphanet was therefore used to estimate the overall prevalence of rare diseases not currently monitored by our registry [50]. The prevalence of the vast majority of the unmonitored entities is unknown or extremely low because these conditions are so rare that they have been described in just a few individuals or families worldwide (Figure 3), and this means that increasing the number of diseases being monitored would not necessarily coincide with a parallel increase in the number of cases being registered. After comparing our own list with the Orphanet list, the overall estimated prevalence of all the diseases not currently monitored by our registry is 3.3/1,000 population, which rises to a maximum of 19.5/1,000, if we include rare tumors. However, a *salami-slicing* effect can be seen for these conditions, that raises the number of entities qualifying as rare diseases, starting from common phenotypic conditions [56]. It can also be argued that incidence is more appropriate than prevalence as an indicator for estimating the burden of cancer at population level [57]. This is because prevalent cancer cases form a very heterogeneous group that – by definition –includes all individuals who have ever been diagnosed with cancer at any time in their life [58], so this group can include people still receiving therapy as well as disease-free survivors, whereas the prevalent cases of other rare diseases are usually individuals living with long-standing chronic conditions.

### Strengths

Despite the above-mentioned limitations, we consider the data presented here of some value because they stem from a good-quality, fully-computerized population-based registry. The monitoring system described here not only provides epidemiological figures for a sizable group of rare diseases that are useful for estimating the magnitude of the problem at population level, it also enables us to establish the relative contribution of different nosological entities to the global burden of rare diseases.

Among others, four elements are fundamental to the successful implementation of this type of registry: the use of a shared web-based system; the feasibility of a modular development of the infrastructure supporting the registry; the "multi-data use" principle; and the registry’s development within the framework of broader health policies addressed to patients with rare diseases.

First, the use of a web-based system simplifies the data collection process and offers economies of scale. The use of a shared infrastructure can promote data collection on ultra-rare diseases, or conditions for which no treatment is currently available, both situations in which a dedicated registry would be difficult to establish and maintain [59]. As demonstrated by the French experience too, involving health professionals from different backgrounds sharing the same information system promotes a multidisciplinary approach to patient care, which is always a challenge, but especially when dealing with complex rare conditions [60,61].

The second element (the modular approach used to develop the information system) ensured a high level of participation in the data collection process, enabling an increasing number of health professionals working in different care network settings to become involved, and facilitating the adoption of the same information system by other Italian regions.

The third element concerns the “multi-data use” principle according to which the data output has to be useful to people who input the data [62]. From the patients’ standpoint, the system ensures a rapid information flow, minimizing the time it takes to obtain benefits, and simplifying the paperwork involved [63]. On the other hand, it enables users to produce on-line statistics based on the data entered and to run searches on all the contents, making the registry a powerful research tool.

Finally, a key issue concerns the registry’s governance. The legally mandatory involvement of governmental bodies (the Regional Health Authorities in the decentralized Italian National Health System scenario) in the setup and maintenance of rare disease monitoring systems guarantees the long-term sustainability of the registration process because it is the first step in the provision of patient care. In addition, as the debate on orphan drug post-marketing surveillance has underscored, the availability of data from independent registries has become very important for the purpose of orienting clinical practice and transparently supporting the decision-making process [64]. In our view, public governance of such registries should be interpreted as evidence of the attention that should be paid to rare disease patients by the community as a whole.

## Conclusions

This study outlines the complex epidemiological profile of rare diseases at population level and emphasizes the usefulness of a monitoring system for the purpose of tackling rare diseases from a global perspective rather than using a piecemeal approach, as recent initiatives in this field have also highlighted [20].

Alongside the debate regarding what value society should attribute to rarity vis-à-vis more common medical conditions, the figures and indicators presented here should dispel any policy-maker’s doubts about allocating resources to rare diseases because they might be perceived as having only a marginal impact on the health of the community.

## Competing interest

The authors declare that they have no competing interests.

## Authors’ contribution

MM and PF conceived the study design, the structure of the manuscript and wrote the initial draft. LVDP performed the statistical analyses. SM, CM and LVDP contributed to the writing of the article and provided feedback. All authors read and approved the final manuscript.

## Supplementary Material

Additional file 1 **List of rare diseases monitored by the Veneto Region’s rare diseases registry.** adapted from the Italian Decree on rare diseases - Annex 1 Reference [28]. Click here for file
